# Intraoperative three-dimensional imaging in ankle syndesmotic reduction

**DOI:** 10.1186/s12891-020-03931-w

**Published:** 2021-01-26

**Authors:** Markus Beck, Manuela Brunk, Alice Wichelhaus, Thomas Mittlmeier, Robert Rotter

**Affiliations:** 1 Department of Orthopedic and Trauma Surgery, St Bernward Hospital Treibstrasse 9, 31139 Hildesheim, Germany; 2 Department of Trauma, Hand and Reconstructive Surgery, c/o Rostock University Medical Center, Schillingallee 35, 18057 Rostock, Germany

## Background

Osteoligamentous injuries to the ankle joint are the most common injuries to the lower limb. The total percentage of fractures of the foot and ankle joint is 56% [1]. Criteria for the choice of therapy are the degree of dislocation and instability of the fracture [2]. Fractures with accompanying unstable syndesmosis injuries are considered a reliable indication for surgery. The incidence of this additional injury in upper ankle fractures is 16% [3]. The accompanying tibiofibular ligament injury significantly worsens the clinical and radiological outcome of the patients [4]. Intra- and postoperative fluoroscopic and radiographic examinations show a very low sensitivity and specificity in the detection of postoperative malpositions of the fibula in the tibial incisura [5–7]. If postoperative malpositions are not detected and the injury heals into a malposition, this will be associated with poor function and premature arthrosis [8].

Postoperative computer tomography is clearly superior to conventional X-rays for the exact assessment of the reduction of the fibula in the tibial incisura [9]. Postoperative CT examinations after ankle osteosynthesis show malpositions of the distal syndesmosis up to 52% [10]. Detected relevant malpositions must be corrected in a second operation.

An alternative to this procedure is the intraoperative use of 3D image intensifiers. These devices allow a multidimensional imaging of the ankle joint [11–13].

The objective of the retrospective single-center study was to assess whether the position of the fibula in the tibial incisura can be determined by intraoperative 3D scanning with sufficient precision. Furthermore, it had to be clarified whether the intraoperative 3D image intensifier examination had an influence on the postoperative revision rate.

## Methods

From September 2007 to December 2015, 1127 patients with fractures of the ankle region were operated at the Rostock University Hospital. Two-hundred of these patients (17.75%) had an instability of the distal tibiofibular syndesmosis. A 3D scan was performed in these 200 patients after reduction and fixation of the fibula in the tibial incisura using one or two syndesmotic screws. Exclusion criteria for the study was an acute or healed contralateral ankle fracture.

All patients were placed on a carbon table in supine position with slightly raised ipsilateral pelvis. The healthy contralateral leg, bent in hip and knee, was placed on a leg shell. After reconstruction and stabilization of the bone injuries, open reduction of the fibula into the tibial incisura and fixation with one or two tricortical syndesmotic screws (3.5 mm small fragment) was performed under conventional radiographic control. The 3D scan was performed before closing the wound. The lower leg was temporary fixed in strain less position with a tape to avoid motion during 3D scan.

The “Vario 3D Image Intensifier” from Ziehm was used. Primarily the “region of interest” and thus the “isocenter” of the 3D scan was determined in the anterior-posterior and in the lateral beam projection by means of fluoroscopy. In order to guarantee a collision-free 3D scan, the isocenter was defined as a radiation-free 135° movement of the C-arm around the injured ankle joint. For definitive imaging, the motorised C-arm performed an orbital movement around the fixed isocenter with a maximum rotation radius of 135° and produced 120 single fluoroscopic images in different angular positions from which the 3D scan was calculated. The three-dimensional data set represents an area with an edge length of 120 mm and allows reconstructions in all three planes (transversal, coronary, sagittal).

The following evaluation parameters for a correct joint position were defined in a standard protocol:

1. Centered position of the fibula in the tibial incisura 10 mm above and at the level of the tibia plafond without overlapping the ventral tibia margin in relation to the width and individual anatomy of the syndesmosis (transversal reconstructions).
2. Equal joint space width fibulotalar and tibiotalar in the joint area (transversal and coronal reconstructions).
3. Congruence of the lateral malleolus joint surface to the lateral talus wall as an indication of correct rotation and length (coronal reconstructions).

All reconstruction planes (coronal, sagittal, axial view) were also used to assess further problems in the form of intra-articular implant positions, relevant joint stages > 2 mm, insufficiently fixed fracture parts in the area of the medial and posterior ankle and intra-articular fragments.

Whenever the 3D scan showed findings requiring correction, immediate correction followed. After position correction of the fibula in the tibial incisura, a new 3D scan was performed in all cases.

The quality of each scan was assessed by the responsible surgeon after viewing. The basis was a purely subjective 4-stage assessment score (good / satisfactory / sufficient / not assessable).

Postoperatively, computer tomography of both ankle joints was performed on all patients. Three distance measurements 10 mm above the joint line were carried out in the postoperative CT. The distance between anterior border of tibial incisura and anterior margin of the fibula, the distance between the middle of the incisura and the nearest point of the fibula, the distance between posterior border of incisura and posterior point of the fibula. A deviation of > 2 mm in comparison to the healthy contralateral side was defined as a malposition of the fibula in the tibial incisura requiring revision.

## Results

A 3D scan was performed on 200 patients with unstable syndesmosis injury during the study period. The mean patient age was 47.4 years (18–83), gender distribution showed 128 (64.0%) male and 72 female (36.0%) patients. Syndesmosis injuries were associated with 120 sole fibula fractures (14 Maisonneuve), 26 isolated inner malleolus fractures, and 30 bi- and 22 trimalleolar fractures. Two patients showed a purely ligamentous injury of the syndesmosis complex. In 190 patients 1, in 10 patients 2 syndesmotic screws were implanted.

One hundred eighty-six of the intraoperative 3D scans (93.0%) showed a correct adjustment of the fibula in the tibial distal incisura according to the surgeon’s assessment.

**Fig. 1:**
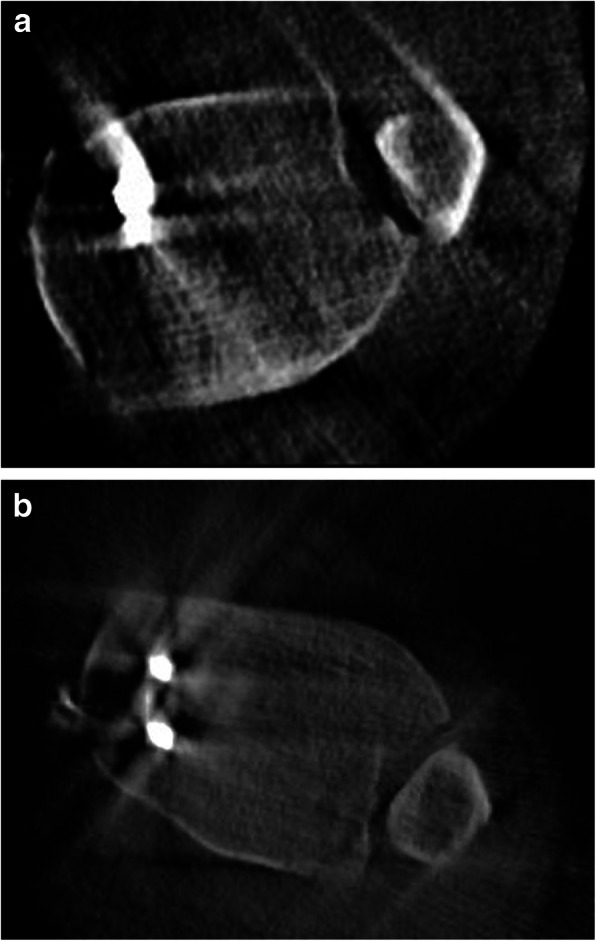
**a** Dorsal malposition of the fibula, axial view. **b** Correct position of the fibula in the tibial incisura after intraoperative correction, axial view

In 14 cases (7%), the fibula position was corrected after intraoperative evaluation of the 3D scan (Fig. 1**a**, 1**b**). A second 3D scan confirmed the successful correction in all cases (*n* = 14).

In 8 patients (4.0%), the 3D scan detected overlong implants and replaced the affected screws with shorter implants.

In 6 patients (3%), the 3D scan showed that medial malleolus or posterior tibia fragments were not sufficiently fixed. Osteosynthesis was then extended to improve stability.

**Fig. 2:**
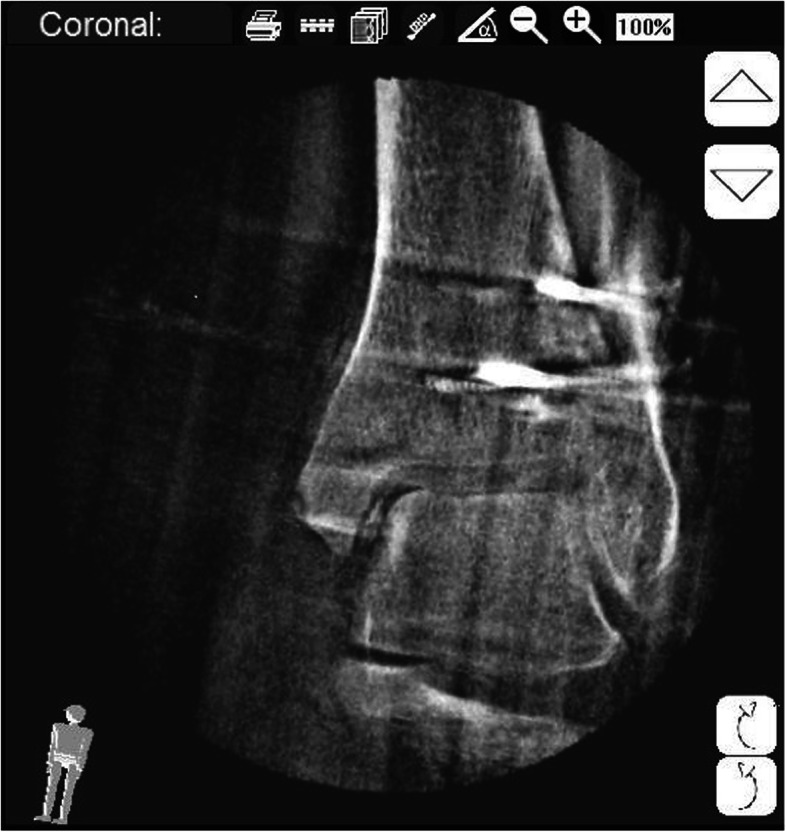
Intraarticular osteochondral fragment medial joint space, coronar view

In 2 patients (1%), an intraarticular osteochondral fragment was detected by the 3D scan, which was not detected by conventional fluoroscopy (Fig. 2). An intraoperative revision of the internal joint space and removal of the fragment were performed.

In summary, 30 of 200 patients (15%) underwent a correction of the surgical restoration based on the intraoperative evaluation of the 3D scan.

In comparison with the healthy contralateral side, evaluations of postoperative CT examinations in no case revealed a defective position of the fibula in the tibial incisura worth revision, a relevant defective position of the joint or an osteosynthesis requiring revision. Hence, the postoperative revision rate was 0%.

The subjective four-stage assessment of the image quality of the 3D scans by the surgeon resulted in 69.3% of the scores being rated “good”, 26.7% as “satisfactory” and 4.0% as “sufficient”. No scan received the evaluation criterion “not assessable”.

## Discussion

Ankle fractures involving the syndesmosis complex generally have a worse prognosis than comparable fractures without tibiofibular ligament injuries. In 347 examined patients, Egol et al. were able to demonstrate that after 12 months the functional outcome and the pain level were significantly worse in the group with syndesmosis injuries [4]. Chissel et al. already reported poor clinical results in 1995 when syndesmosis width after surgical treatment exceeded the radiologically measured value of > 1.5 mm [14]. Andersen noted a difference of more than 2 mm of the sagittal anterior tibiofibular distance as a predictor for poorer clinical outcome [15]. Moreover, Leeds and Ehrlich proved a significant correlation between arthrosis development and accompanying syndesmosis injury [8]. Current medium-term study results obtained by Veen et al. confirm a significantly higher arthrosis rate associated with ankle fractures with syndesmosis injury [16]. In addition, Ovaska et al. were able to show that, at 59%, malrepositioned syndesmosis is the most frequent cause of revision surgery of ankle fractures [17].

The intraoperative malposition rate of the distal tibiofibular syndesmosis in closed reduction is up to 52% and can be reduced to 15% by open reduction of the fibula with direct visualization of the syndesmosis region [3, 18]. However, also malposition rates after open reduction are still high and require a reliable position control of the distal syndesmosis region. All conventional X-ray parameters (tibiofibular clear space, tibiofibular overlap, etc.) do not allow a sufficient assessment of the fibula position to the tibia [5, 6, 19].

This applies to all syndesmosis injuries since Franke et al. could not identify risk factors such as injury type or fracture morphology after analyzing 251 patients with syndesmosis injuries, which are associated with a lower rate of syndesmosis malposition [20].

Relevant evaluation criteria are the position of the fibula in the tibial incisura and the rotation of the fibula considering correct length reconstruction [9]. CT-Measurements 10 mm above the tibial plafond taking into consideration the diastasis and anterior-posterior translation of the fibula were found to be parameters with high interobserver and intraobserver reliability [21].

The position of the foot, according to the studies by Levack and Vetter, has no relevant influence on the tibiofibular distance nor on the tibiofibular angle. Therefore, the intraoperative scan can be performed in any position of the ankle and foot [21, 22].

Multidimensional intraoperative imaging of the syndesmosis region is also possible using 3D image intensifiers. A small case series of 10 patients with syndesmosis injuries was presented by Ruan et al. in 2011 [23]. An intraoperative 3D scan was performed before positioning the adjusting screw with the joint being temporarily adjusted by means of reduction forceps. The measurement parameter was the distance to the anterior and posterior facets of the tibia. The aim was to achieve equal measuring distances. Once fine correction and adjusting screw application had been completed, a final second 3D scan was performed. In all cases, this scan showed a central and symmetrical positioning of the fibula in the tibial incisura.

Summers et al. reported a lower rate of 5.5% (1/18 patients) malreduction in syndesmotic injuries shown by intraoperative 3D scan [24]. They used conventional X-ray settings of the uninjured side as a template to assess the reduction before the intraoperative 3D scan. They concluded that intraoperative CT is only necessary in cases where conventional radiologic signs didn’t indicate an accurate restoration The results of the small case study do not match the results of many other studies in which a significantly higher rate of malreduction was detected.

Moon et al. reported a significant higher intraoperative revision rate of 23,1% using a 3D image intensifier for ankle fractures with syndesmosis injuries [25].

Franke et al. performed intraoperative 3D scans in 251 consecutive patients with syndesmosis injuries after adjusting screw placement, which resulted in direct intraoperative correction of osteosynthesis in 32.7% of patients [26]. The main reason was a malposition of the fibula in the tibial incisura in 25.5% and a necessary correction of the fracture reduction in 5.2% of the patients. Corrections due to implant misalignments were necessary in 2% of patients.

Davidovitch et al. compared the conventional versus 3D scan controlled intraoperative reduction of the ankle joint in 36 patients [27]. In the relevant measuring range of 2 mm difference, significantly more postoperative malpositions in the control CT were found in the conventionally radiologically controlled group. Our own data showed a lower malpositioning and correction rate of 7% which may be explained by the generally direct visualization of the syndesmosis stabilisation. Another surgical parameter that significantly influences the reduction result is the positioning of the reduction forceps in the anterior third of the tibia [28].

A problem that has not yet been finally resolved is the correct assessment of the syndesmosis region. The common parameters used are the fibular length, fibular position and rotation in the incisur [9]. However, the tibial incision has a large anatomical variance in shape. Seventy-five percent of the incisions have a concave shape, 16% a convex shape and 8% are not typable [29]. Elgafy et al. found 67% convex and 33% flat angled incisures 9–12 mm above the tibial plafonds [30]. This makes reliable rotation measurement difficult in the absence of normal values ​​and uncertainty about the best measurement method at the level of the syndesmosis 10 mm above the ankle joint. Knops, for example, compared the reliability and accuracy of 4 measurement methods using a 3D rotational C intensifier [31]. Two of these measurement methods were difficult to carry out and even the best method, measuring the angle between the tangent of the anterior tibia surface and the bisection of the vertical midline, was only fairly reliable and accurate.

Comparison of the healthy opposite side is therefore seen as the gold standard for assessment in the CT. Schon summarized the results of 16 CT studies that carried out a total of 35 different measurement methods [32]. The study demonstrated low native side-to-side symmetry. Furthermore, there is no single measurement method that adequately captures the complexity of the possible misalignments. At least 3 different measurement methods are necessary to record the relevant criteria of translation medial / lateral, anterior / posterior and fibular rotation. In particular, the sole qualitative side-by-side comparison without measurement data collection shows a very low level of intra- and interobserver reliability and should not be used as an assessment parameter [33].

The healthy opposite side for comparison is not available with the intraoperative cone beam CT because the imaging volume is too small. A second scan of the healthy opposite side would have to be performed. In terms of radiation protection and the additional expenditure of time, it must be assessed critically and this approach has not yet been investigated in studies. Complex intraoperative measurements of the rotation and translation of the fibula in the tibial incision are also not expedient because of the lack of normal values, the large anatomical variance and the lack of comparison with the contralateral side [29, 30, 32].

It is therefore important to define comprehensible criteria in order to be able to assess the intraoperative cone beam ct examinations. The criteria we have described correspond to the assessment parameters defined by Franke and, more recently, by Vetter [26, 34]. The fibula should be symmetrical in the incisure 10 mm above the tibial joint line, and the arch between the anterior margin of the fibula and the tibia should be harmoniously elliptoid. There must be an equal fibulotalar and tibiotalar clear space in coronal and sagittal

view. The fibular length needs also be assessed.

The intraoperative measurement of the fibula rotation has proven to be impractical at the level of the syndesmosis. The rotation measurement below the ankle joint line seems to be easier. The joint-side corticales of the malleoli are used as reference. Vetter et al. found the area 4–6 mm below the talar joint line to be the ideal measurement point for fibular rotation in 100 healthy joints [35]. The mean angle was 8.4 ° +/− 4.9. However, the absolute values ​​varied between 0 and 26.

Compared to CT examinations, intraoperative radiation exposure resulting from the 3D scan can be classified as very low in total. Beerekamp et al. reported a maximum dose of 17 μSV for a 3D extremities scan compared to a 200 μSV dose for a postoperative CT examination [36].

## Conclusion

The results of our study confirm that an 3D image intensifier examination allows a reliable intraoperative assessment of the anatomy of the distal syndesmosis region and the reconstructed ankle joint. Malpositions of the fibula in the tibial incisura and defective osteosyntheses were reliably detected and corrected intraoperatively. According to our data, a routine postoperative CT examination of the region is dispensable if the 3D scan can be easily assessed.

## Data Availability

The datasets used and/or analyzed during the current study are available from the corresponding author on reasonable request.
